# A Contribution to the Study of Colles Fracture

**Published:** 1887-12

**Authors:** D. M. Totman

**Affiliations:** Syracuse, N. Y.


### *A CONTRIBUTION TO THE STUDY OF COLLES FRACTURE.*

By D. M. TOTMAN, M. D., Syracuse, N. Y.

On the morning of November 3, 1887, J. W., aged fifty-five, a strong, muscular man, was at work upon a flat roof. Being near the edge, and possibly forgetting his position, he rose, and stepping backwards, fell a distance of thirty feet to the ground.

He was admitted to St. Joseph's Hospital, and, among other injuries, had a well-marked colles fracture of the left wrist. On account of profound collapse, only a slight attempt at reduction was made. The patient died on the following morning. Examination of the arm was made twenty-eight hours after death. By a careful dissection over the posterior aspect of the ulna, it was found that the tendon of the extensor carpi ulnaris was displaced. The styloid process of the ulna was fractured, and the portion broken off was left attached to the annular ligament of the wrist-joint.

Only a small amount of manipulation was used, but sufficient to show that extension alone would not replace the dislocated tendon. There was a transverse, non-impacted fracture of the lower extremity of the radius.

